# The Mechanical Microenvironment in Breast Cancer

**DOI:** 10.3390/cancers12061452

**Published:** 2020-06-03

**Authors:** Stephen J.P. Pratt, Rachel M. Lee, Stuart S. Martin

**Affiliations:** 1 Program in Biochemistry and Molecular Biology, Department of Physiology, and Marlene and Stewart Greenebaum NCI Cancer Center, University of Maryland School of Medicine, 655 W. Baltimore Street, Bressler Research Building, Rm 10-020 D, Baltimore, MD 21201, USA; 2 Department of Physiology and Marlene and Stewart Greenebaum NCI Cancer Center, University of Maryland School of Medicine, 655 W. Baltimore Street, Bressler Research Building, Rm 10-020 D, Baltimore, MD 21201, USA; RaLee@som.umaryland.edu

**Keywords:** mechanosensation, mechanotransduction, mechanoresponsiveness, mechanobiology, breast cancer, stiffness, interstitial fluid pressure, solid stress

## Abstract

Mechanotransduction is the interpretation of physical cues by cells through mechanosensation mechanisms that elegantly translate mechanical stimuli into biochemical signaling pathways. While mechanical stress and their resulting cellular responses occur in normal physiologic contexts, there are a variety of cancer-associated physical cues present in the tumor microenvironment that are pathological in breast cancer. Mechanistic in vitro data and in vivo evidence currently support three mechanical stressors as mechanical modifiers in breast cancer that will be the focus of this review: stiffness, interstitial fluid pressure, and solid stress. Increases in stiffness, interstitial fluid pressure, and solid stress are thought to promote malignant phenotypes in normal breast epithelial cells, as well as exacerbate malignant phenotypes in breast cancer cells.

## 1. Introduction to Mechanical Signaling

Biophysics is a field of research that endeavors to understand biology from the standpoint of physics, by using physics-based theories and methods, which includes the physical/mechanical properties of cells and surrounding tissue (e.g., whether they are rigid or pliable). Mechanobiology is the study of the interaction between physical forces and cells, or even single biomolecules (e.g., proteins), and how mechanical cues alter cellular responses. Forces acting externally on cells (i.e., mechanical stimuli) include stretch, compression, tension, shear stress, and substrate rigidity or deflection. Through mechanosensation, cells sense changes in the prevailing intracellular and/or extracellular mechanical homeostasis on a molecular level, which ultimately initiates intracellular signaling. This translation of physical signals acting on cells to a cellular response via intracellular signaling is known as mechanotransduction.

This review on mechanobiology and mechanical signaling will begin at the junction between mechanical stimulus and cellular force-sensing mechanisms (i.e., mechanosensation), including the role of individual proteins and protein complexes. The resulting conversion of mechanical stimuli into electrical and chemical signals (i.e., mechanotransduction) that initiate a cell’s ultimate response will also be outlined. Furthermore, the bidirectional interaction between cells and their mechanical microenvironment, known as mechanoreciprocity, is considered. Finally, these ideas converge on the notion that the cell’s mechanical microenvironment and mechanotransduction signaling are dysregulated in breast cancer, which promotes cancer cell malignant phenotypes and tumor growth.

## 2. Mechanoresponsiveness: Mechanosensation and Mechanotransduction

One classic example to introduce mechanosensation and mechanotransduction is hearing (reviewed in [1]). As sound waves enter the ear, they eventually reach the inner hair cells. Sound wave vibrations interact with clusters of hair cell stereocilia (actin-based protrusions from the cell into the extracellular space) and deflect them. About 1µm of deflection is needed for the maximal mechanical opening of transduction channels at the tip of the stereocilia [2]. This force transmission from vibration to stereocilia deflection allows calcium ions (Ca^2+^) to enter, which electrically depolarizes the hair cell, and initiates neurotransmitter release and the activation of neurons connected to the hair cells by synapses. Therefore, sound wave mechanical stimuli were translated into brain signals that process hearing. The mechanism for mechanosensation is the stereocilia deflection followed by ion channel activation, while mechanotransduction is mediated by ion flux, electrical depolarization of the cell, and neurotransmitter release. Many other cell types are also sensitive to a variety of mechanical stimuli, such as osteocytes to laminar fluid flow [3], cardiomyocytes to stretch [4], kidney epithelial cells to flow [5,6] or cilia bending [7], platelets to substrate stiffness [8], endothelial cells to disturbed fluid flow [9], hepatocytes to stiffness [10], T cells to antigen catch bond binding [11,12], adipocytes to stretch [13], and chondrocytes to osmotic stress [14]. Furthermore, many cell processes are sensitive to mechanical stimuli, such as glycolysis to substrate rigidity [15], epithelial-to-mesenchymal transition to matrix stiffness [16], cell division to stretch [17], cell invasion to matrix deflection [18], and gene activation to fluid flow [19]. The variety of mechanically-stimulated biological responses in different cell types and the conservation across organisms (reviewed in [20]) suggests that evolution has continued to incorporate the prevailing physical aspects of our world over time. 

**Figure 1:**
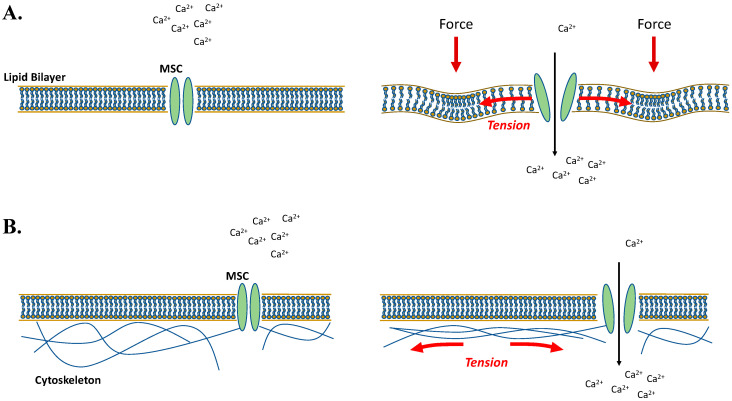
Models for activation of mechanically sensitive ion channels. Two prevailing mechanisms for the transmission of forces to mechanosensitive ion channels (MSC) for gating are (**A**) tension in the lipid membrane and (**B**) tension in the cytoskeleton. **A:** Force transmission from exogenous mechanical stimuli create tension in the cell membrane that is transmitted to ion channels resulting in channel gating and ion flux. The bacterial MscL, MscS, MscM channels and mammalian Piezo channels are gated directly by membrane tension. **B:** Alternatively, for ion channels directly linked to the cytoskeleton, gating and ion flux can result from the transmission of tension in the cytoskeleton to MSCs, such as the drosophila TRP channel NOMPC.

The first major mechanosensors discussed are mechanically activated ion channels, also called mechanically sensitive (MS) ion channels, which are found in bacteria, plants, and mammals. Ion channels are membrane bound proteins that form a central pore that can pass (i.e., conduct) ions when activated and open, or block ion flux when inactivated or deactivated and closed. Ion channels are gated (opened/closed) by a variety of mechanisms such as voltage, ligands, and temperature, however, MS channels are also gated by forces such as tension. It should be noted that while in vitro studies have yielded important information on force driven gating mechanisms, it is still unclear exactly how macroscopic forces acting on cells in vivo (e.g., stretch, compression, tension, shear stress, and ECM rigidity) are transmitted to MS channels. With that in mind, two prevailing mechanisms for the transmission of forces to channels for gating are tension in the lipid membrane and tension in the cytoskeleton (Figure 1).

Structure and modeling studies on bacterial MS channels (MscL, MscS, MscM) suggest that these channels are gated by ~10 mN/m of tension in the lipid membrane [21,22,23], which tilts the channel’s transmembrane α-helicies by ~25°–30° [24,25,26], resulting in an increase in pore region diameter of ~16 angstroms [27], allowing ions to flow (Figure 1A). Another major group of MS channels are mammalian Piezo proteins and these are also thought to be gated via tension in cell membranes. Structure studies of Piezo 1 [28,29] and Piezo 2 [30] reveal that these MS channels are made of a 3-blade propeller-like structure which is embedded in the cell membrane. Tension in the membrane is thought to move these blades in a downward fashion, thus transferring conformational changes to the inner pore regions and opening the pore to allow ion flux. The cap region, which is involved in channel inactivation [31], may also function as part of the gate or serve as one of a dual gating mechanism [30]. Electrophysiological studies of Piezo channels show that they can be truly gated by membrane tension [32] without any contribution from the underlying cytoskeleton [23], however, unlike bacterial MS channels, these seem to be gated by a lower tension threshold (~5 mN/m) [23]. Furthermore, it is possible that Piezo channels can be activated by either membrane or cytoskeletal tension and more studies are needed to determine the complete functional mechanism. Of additional note is that voltage may also play a role in addition to tension, as Piezo 1 displays a voltage-dependent inactivation [31]. Piezo channels are selective for cations over anions via a negatively charged DEEED protein sequence in the pore region [28], but they are relatively non-selective between cations. Piezo channel pore regions are permeable to both monovalent ions (K^+^, Na^+^, Cs^+^, Li^+^) and most divalent ions (Ba^2+^, Ca^2+^, Mg^2+^) [33]. Despite the non-selectivity, most studies focus on Piezo-dependent Ca^2+^ ion flux and downstream signaling. Other relevant mechanically activated channels are some members from the transient receptor potential (TRP) family of proteins. The TRP family of ion channels are composed of seven subfamilies: TRPC (canonical), TRPV (vanilloid), TRPM (melastatin), TRPP (polycystin), TRPML (mucolipin), TRPA (ankyrin) and TRPN (NOMPC-like) (reviewed in [34]). TRP channels range in selectivity, but are generally permeable to Ca^2+^ and Mg^2+^ ions, and can be activated in response to cold/menthol, stretch-activation, pH, Ca^2+^, and voltage (reviewed in [34,35]). There are many data supporting that some of these channels are activated by mechanical stimuli, however, TRP channels are still not considered definitive MS channels (i.e., directly gated by forces), as has been demonstrated for bacterial MS channels and Piezo proteins. Indeed, many TRP channels are insensitive to direct membrane stretch [36], as demonstrated using patch clamp recordings of single channel activity coupled to pipette suction in cells and artificial membrane bilayers (channels studied: TRPC3, TRPC5, TRPC6, TRPM4, TRPM8, TRPV1, TRPV3, TRPV4, TRPA1, PKD2L1 and TRPML1). However, the direct gating via stretch/membrane tension demonstrated for Msc and piezo channels is not necessarily a prerequisite for determining that TRP channels are mechanically dependent. In other words, TRP channels can be indirectly mechanically activated and still function in mechanically-stimulated signaling pathways [37]; they simply would not serve as the mechanosensor in the pathway but rather remain downstream of mechanosensors. Examples of TRP channels involved in mechanically-stimulated signaling include the well-studied TRPV4 which can be activated by hypotonicity (i.e., osmotic stimulation) [38], fluid flow [39], and tension [40]. Other examples include TRPM4 [41], TRPM7 [42], TRPC1 [43], and TRPC6 [44]. Finally, some channels in the TRP family do seem to be mechanosensors, but support the cytoskeleton tension model for activation (Figure 1B). For example, microtubules and the microtubule-associating ankyrin repeat domains in the drosophila TRP channel NOMPC are necessary for mechanogating [45]. TRPV4 also supports a tether-based mechanism for transferring cytoskeletal tension to MS channels, since direct application of force to integrins and cytoskeletal strain rapidly activates TRPV4 Ca^2+^ influx [46]. Finally, it is worth mentioning the K^+^ channels TRAAK and TREK1, that are directly mechanically sensitive and gated via lipid membrane tension [47,48].

**Figure 2:**
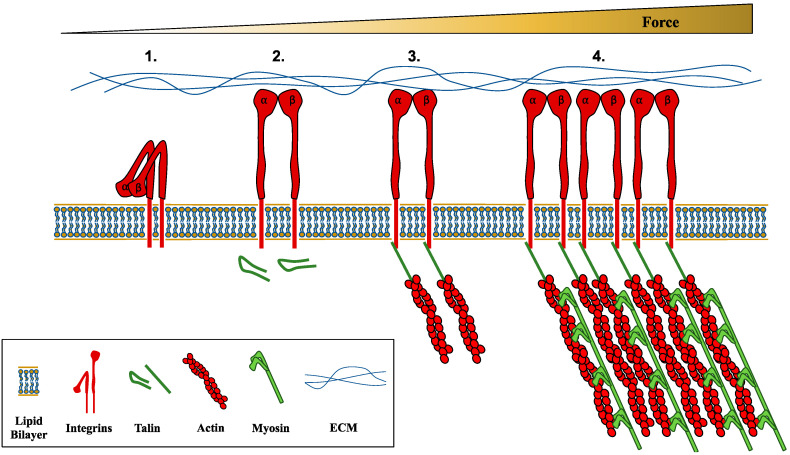
Integrins as force-dependent mechanosensors. Left to right with increasing force: **1**. Tensile forces can extend ⍺/β integrins from a downward-bent fashion to a thermodynamically unfavorable extended-open confirmation. **2**. Force-induced catch-bond formation with ECM prolongs bond lifetime. **3**. Force-induced conformational changes to the intracellular adaptor protein talin, lock talin in an unfolded state and increases adhesion growth. **4**. Finally, force can increase integrin clustering, leading to mature integrin adhesions.

In addition to ion channels, other major mechanosensors are cell adhesion transmembrane receptors: integrins (reviewed in [49,50,51]) and E-cadherins (reviewed in [52,53]), which are key components of their respective cellular structures (focal adhesions/invadosomes and adherens junctions). While desomosomes have not been described in as much detail as integrins and E-cadherins, they are worth mentioning, as some recent work supports a role for mechanosensation (integrins, E-cadherins, and desmosomes are part of the focus of this review, however, it should be noted that there are many other adhesion molecules such as N-cadherins, P-cadherins, and selectins). Integrins connect cells to substrates such as ECM, while E-cadherins and desmosomes enable cell-cell connections. Integrins are heterodimeric transmembrane proteins made of various combinations of α and β subunits (from 18 α types and 8 β types), which underlie the ability to bind to different extracellular ECM components (glycoproteins (fibronectin, laminin), fibrous proteins (collagen, elastin), and proteogylcans (perlecan, hyaluronan)). Integrins are also bound intracellularly to the actin cytoskeleton via adaptor proteins such as talin and kindlin. The necessity of these cytosolic adaptor proteins for integrin activation gives rise to the inside-out signaling model for integrin activation (i.e. intracellular factors mediate integrin activation). However, integrin activation is also force-dependent, giving rise to an outside-in signaling model for integrin activation (i.e., extracelluar factors mediate integrin activation), and therefore force-dependent integrins act as mechanosensors (Figure 2). Integrins have been undoubtedly viewed as adhesion receptors activated through their extracellular and intracellular connections, however some recent evidence suggests that the integrin receptors themselves may instead (or possibly also) activate through changes in lipid membrane tension [54]. Integrins are mechanically sensitive in several ways: integrin activation/bond formation, bond strengthening (catch-bond, i.e., where applied forces strengthen a bond [51]), and clustering/maturation. Integrins activate when they move from a downward-bent fashion to a thermodynamically unfavorable extended-open confirmation protein, a conformational change that can be induced via applied tensile forces [55,56,57]. High-affinity binding to ECM occurs during the extended-open confirmation, however, force-induced catch-bond formation (10–30 pN [58]) can prolong bond lifetime. While talin is also necessary for integrin activation, talin itself is mechanically sensitive. Talin proteins can fold and unfold via pN range forces [59], which leads to the exposure of cryptic vinculin binding sites [60], locking talin in an unfolded state [61], and increasing adhesion growth [62]. Finally, force can increase integrin clustering [63,64], leading to mature integrin adhesions [65]. Ultimately, integrin activation, the recruitment of intracellular adaptor proteins, and the formation of focal adhesions lead to increased cellular contractility and intracellular force generation, through the binding of myosin and actin proteins (Figure 2), which can lead to cell signaling and gene expression. Actomyosin contractility is necessary for maintaining cell shape, collective cell migration, cell division, and metabolism among other functions [66,67,68]. Importantly for cancer, actomyosin contractility can aid in enhancing genomic instability [69], tissue stiffness and tumor growth [70], and cancer cell invasion [71]. Moreover, integrins play a role in promoting every step of the metastatic cascade [72], and metastasis can be affected by different integrin subtypes. For example, integrin α2 promotes melanoma metastasis to the liver [73], while integrin α3β1 aids in breast cancer lung colonization [74] and αvβ3 promotes bone metastasis [75].

E-cadherins are also mechanically sensitive. E-cadherin proteins form cell-cell connections when each cell’s respective cadherins are bound to one another extracellularly. The respective E-cadherins are also intracellularly bound by p120-catenin, β-catenin, and α-catenin. E-cadherins are major proteins downregulated in cancer cells through a mechanism known as epithelial-to-mesenchymal transition (EMT). This downregulation is thought to be a major mechanism by which epithelial cancers acquire the ability to migrate and invade away from primary tumors and progress through metastasis. E-cadherins are sensitive to tensile forces which can tune the type of bond strength between them, such as long-lived catch bonds and short-lived slip bonds, and thus bond strength between cells [76,77]. Furthermore, the E-cadherin/β-catenin/α-catenin complex binds actin under force via catch-bonds [78,79]. α-catenin seems to be a major force sensor in the E-cadherin/β-catenin/α-catenin complex that facilitates adhesion strengthening. In a force dependent manner, α-catenin changes confirmation that allows for the subsequent binding of vinculin [80], through the exposure of cryptic vinculin binding sites [81]. Moreover, the unfolding of α-catenin and subsequent vinculin binding can occur under 5 pN applied force, while vinculin binding stabilizes the open α-catenin conformation [81]. Vinculin can then bind actin, again in a force dependent manner, via catch bond formation [82]. This ultimately increases adhesion strength [83]. Force generated by cell-cell contacts can reach 100 nN [84].

The third set of adhesion complexes mentioned are desmosomes. Desmosomes mediate cell-cell connections much like E-cadherins, however they internally bind to intermediate filaments instead of actin. Future work might detail desmosome mechanosensation much like that of integrin or E-cadherin, but these mechanisms are not yet firmly established for desmosomes. At least one study using FRET-based sensors show that, while desmosomes do not experience mechanical load under homeostatic conditions, they do so under applied tension [85]. Interestingly, this also depends on the orientation of applied force (i.e., perpendicular vs. parallel). The authors suggest that the data support a stress absorbing function for desmosomes. 

Mechanotransduction signaling pathways typically follow mechanosensation. The most immediate responses to the mechanosensation mechanisms described above (mechanically-dependent ion channel gating, integrin activation and talin unfolding, E-cadherin bond strengthening and α-catenin unfolding) are intracellular biochemical and electrical signaling: ion flux, vinculin binding/integrin clustering, and vinculin binding/adhesion strengthening (i.e., mechanotransduction mechanisms). However, mechanotransduction pathways have also been detailed further downstream from mechanosensors. Ca^2+^ ion flux via mechanically activated piezo channels can theoretically stimulate a large variety of Ca^2+^ sensitive effectors, enzymes, and transcription factors that ultimately converge on cell functions (reviewed in [86]). For example, force stimulated Ca^2+^ flux through piezo channels can stimulate vasculature architectural organization and cell polarity [87] or epithelial cell division [17]. Piezo specific Ca^2+^ signaling can also drive stem cell migration [88], the formation of focal adhesions [89], or cell cycle progression [90]. For integrins, forces via increased substrate stiffness can lead to the formation of integrin and focal adhesion kinase rich mature cell adhesions [62]. These focal adhesions mechanically guide cell migration through force sensing [91]. Tensional force on integrins can also stimulate guanine nucleotide exchange factors, Src family tyrosine kinases, and FAK/Ras/ERK signaling [92]. Furthermore, mechanically activated integrins or polymerized actin can activate transcription factors YAP [62] and MRTF [93], respectively. The mechanical activation of transcription factors such as MRTF via integrin receptors and actin polymerization is thought to arise from force translation to nuclear proteins linked to actin [94], such as SUN and nesprin of the LINC (linker of nucleoskeleton and cytoskeleton) complex. The idea that mechanotransduction of the nuclear membrane and chromatin, mediated through integrins and actin, and the idea that it can activate gene expression, has been reviewed [95,96]. E-cadherin adhesions are imperative to maintaining epithelial barriers and also lead to downstream mechanotransduction signaling. Interestingly, force induced vinculin recruitment at the E-cadherin/β-catenin/α-catenin complex was shown to stabilize cell junctions in junction-poor cancer cells [97], a finding with intriguing implications for cancer metastasis. Moreover, the mechanics of cell-cell adhesions in single and collective cell migration have been extensively studied (see reviews [98,99]).

## 3. Mechanoreciprocity

Sensation of the surrounding mechanical environment and resulting mechanotransduction responses represent a one-way directional signal from environment to the cell, however cells also produce signals that impact their environment. This bi-directional signaling between cell and environment (or even cell to cell) is termed dynamic reciprocity [100,101,102,103] or mechanoreciprocity [104,105]. Mechanoreciprocity has been defined as a process by which cells within a tissue “that become mechanically challenged must respond to the exogenous force by reciprocally exerting a proportional cell-generated force” [104], but more recently, mechanoreciprocity has been broadened to describe “a cyclic process in which cells modify the organization and elastic response of the environment and reciprocally adjust their behavior” [105]. Demonstrations of cell-ECM mechanoreciprocity come from studies using cells plated on substrates of different stiffness, as a means to apply a range of forces to cells and measure cell response, where cells were found to reciprocate forces and also modify their substrates. Cells exert stress on their substrates which deform, densify, stiffen, and align ECM [106,107,108,109]. Cells, in turn, respond to environmental stiffness with increased force generation and cell stiffening [106]. Indeed, cells can tune their stiffness to match the rigidities of their substrates [110] and, interestingly, substrate stiffness can guide preferential cell migration toward stiffer substrates [111,112,113]. Alternatively, stiffer 3D matrices can induce cells to contract their surrounding matrix and increase local collagen density, which in turn results in mature focal adhesion formation [114]. Other studies show that ECM alignment from cell-generated forces enhances and directs cell migration [108,115]. Other examples of mechanoreciprocity mechanisms come from cell migration studies in 3D matrices. ECM can mechanically constrain cell migration depending on the porosity of the matrix [116]; cells cannot pass through small pores due to limitations in nuclear deformability. Cells respond to these mechanical limitations with active degradation of the matrix to widen the pore size and the subsequent sensing of decreased spatial constriction allows cells to resume migration [116,117].

Thus, there can be a cyclical mechanical interplay between environmental cues and cell adaptation responses. Cells can sense the mechanical characteristics of their environment, and they can then respond by altering their mechanical/functional state or by modifying the environment, but they can also subsequently respond to the environment they modified. These interactions are normal under proper contexts, but can become pathological if the mechanical interplay fails to reach steady state. In the context of breast cancer chronic remodeling of the mechanical microenvironment, especially that of substrate/ECM, this promotes malignant cell functions and tumor formation [118,119].

## 4. Mechanical Forces and Signaling in the Normal Breast

There are many excellent examples by which in vitro experiments are able to apply mechanical stimuli to cells which closely parallel mechanical stimuli experienced by cells and tissues in vivo. By precisely mimicking in vivo mechanical stimuli, researchers can achieve not only a better understanding of how mechanosensation and mechanotransduction processes are integrated by cells and tissues in vivo, but also determine the physiological or pathological outcomes. The details of pathological responses to mechanical stimuli can then be leveraged for identifying therapeutic targets for disease. For example, cardiomyocytes that make up the heart experience about 8% stretch in vivo during diastolic ventricle dilation [120]. Researchers model this in a dish by briefly (~10 s) stretching single cardiomyocytes 8% of cell length to initiate Ca^2+^ signaling [4,121], and have further discovered the dysregulation of mechanically-activated Ca^2+^ in muscular dystrophy [4,122]. Similarly, bone osteocytes experience fluid shear stress in vivo when bones are mechanically loaded [123,124]. This fluid flow is experimentally replicated in vitro [125] and fluid shear stress at 4 dynes/cm^2^ results in the downregulation of a protein inhibitor of bone formation in osteocytes [3]. These clear connections between in vivo mechanical stimuli and in vitro modeling illustrate logical approaches to mechanobiology research.

Demonstrations of dynamic mechanical stresses such as fluid flow or cell stretch in the breast have less precedent than in bone and muscle. In a review by Paszek and Weaver [104], the authors suggest that the mammary gland is subjected to acute forces at different stages. They infer from examples of physical forces in embryonic development [126,127] that forces likely also drive mammary development and branching morphogenesis. They (as have others [128,129]) discuss other sources of mechanical stress, such as during lactation (e.g., offspring suckling, myoepithelium contraction on underlying lumnial epithelial cells to squeeze milk from ducts, fluid flow from milk delivery, and compressive forces from milk build up), or during involution (i.e., initial engorgement of the gland with milk) [130,131]. Moreover, the massive remodeling of tissue during pregnancy and involution [132] may be sources of cell proliferation related solid stress, as has been shown for tumors [133]. While logically sound, these sources of mechanical stress in vivo are without sufficient supporting empirical data to quantify the magnitude of applied stresses.

In contrast to the multi-developmental states of the mammary gland (puberty, pregnancy, lactation, and involution), Paszek and Weaver view the resting mammary gland as mechanically static [134]. On the contrary, Gefen and Dilmoney attempt to describe the daily mechanical loads on the breast [135] and compare static postures (standing, prone, and supine) and dynamic motions (running, stair climbing, and jumping), by modeling forces based on known values from the literature (similar sources of mechanical stimuli are also mentioned briefly in a review by Schedin and Keely [129]). They ultimately conclude “it is reasonable to assume that the healthy breast is subjected to cyclical forces with peaks of 5–15 N, 5000 times per day or ~2,000,000 times per year of normal activity”, however this study is largely theoretical. Unfortunately, the physiologically relevant acute or dynamic forces and mechanical stimuli experienced by mammary cells and tissue in vivo seem to be largely a point of discussion due to the lack of direct measurements, and this thus remains unclear (a view shared by the Bissell group [128]). While this imparts a major challenge to researchers attempting to study and model physiologically relevant mechanosensation and mechanotransduction mechanisms of the breast, it also means that, because mammary biophysics is incompletely defined, there are many unexplored questions available for researchers to investigate.

Despite the ambiguities surrounding acute forces dynamically exerted on the mammary gland in vivo, human mammary epithelial cells are indeed mechanically sensitive to transiently applied stimuli in vitro. In response to touch by micropipette, primary mouse mammary epithelial cells respond within seconds through changes in membrane potential [136] and intracellular Ca^2+^ concentrations [137,138,139,140]. Other studies show that non-tumorigenic HC11 mammary cells respond to 15 min of cell-stretch with ERK1/2 and STAT3 phosphorylation [131] and MCF10A cells respond to 2.5 dynes/cm^2^ of fluid flow shear stress with AMPK activation within minutes [141]. Extended mechanical stimulation can also lead to a response, such as MCF10A cells embedded in 3D matrix which respond to 4 h of fluid flow at 4.6 μm/s (∼1 Pa (1 pN/μm^2^) average stress), with accumulation of actin and vinculin upstream of the flow [142]. We can infer from these proof of concept examples of mammary cell mechanoresponsiveness to transiently applied mechanical stimuli, that it is likely that these cells experience and respond to dynamic mechanical forces in vivo. That these mechanisms have been evolutionarily preserved also suggests an in vivo functional purpose. One possible function is to preserve epithelial barrier homeostasis. This idea is supported by one recent study in MCF10A cells, showing that inhibition of mechanically sensitive channels or downstream Ca^2+^-sensitive CAMKK2 disrupts epithelial sheet integrity and the induction of epithelial-to-mesenchymal transition markers [143]. The implications for epithelial carcinomas that account for 90% of human solid tumors are intriguing [144]. Future work may reveal direct evidence for fast-acting mechanical stimuli and functional responses in vivo, such as fluid flow or cell stretch/compression, but without clear experimental precedent, researchers should remain aware of the limitations of their mechanical models.

In contrast to the more traditionally viewed acute and dynamic mechanical stresses discussed above, the relationship between the mammary gland and ECM has been described in much more detail, and ECM is viewed as both a mechanical and biochemical signal important in mammary development and maintenance (reviewed in [100,101,103,104,129]). Early indications that ECM mechanical signaling plays a role in normal mammary physiology were demonstrated in vitro using cells plated in 3D collagen environments of different stiffness, which were independent of collagen concentration. Mammary epithelial cells were able to differentiate into duct-like tubules and secrete milk proteins when cultured in floating collagen gels (softer), but not on culture dish-attached collagen gels (stiffer) [145,146,147,148]. Mechanistically, this compliant substrate-dependent tubulogenesis seems to require cell contraction mechanosensing [149]. These works established a mechanical role for ECM in normal mammary epithelial cell physiology and showed that mechanical signaling was necessary for proper function. As such, the mechanical relationship between breast epithelial cells and ECM largely account for how breast mechanobiology is modeled in vitro, and the intimate relationship between the mammary gland and ECM in vivo justifies such a model.

Of note, in vitro studies on ECM mechanics and mammary physiology so far mainly rely on delayed (i.e., measuring responses after hours to days in cell culture) and fixed-timepoint experimentation (i.e., fixed immunofluorescence, Western blots) without sufficient temporal resolution to fully determine mechanism and molecular details. Therefore, the precise dynamic mechanical nature of ECM-mammary interaction remains unclear for both in vitro and in vivo settings. Dynamic cell-ECM mechanical interactions that have been established in other cell types in vitro suggest that similar mechanisms are possible for mammary epithelial cells. For example, bond dissociation between the major cell connection to ECM, integrin receptors, and the ECM protein fibronectin occur within seconds as a function of force [62]. Focal adhesion force fluctuations (i.e., tugging) between mouse embryonic fibroblasts and ECM also occur on a second time scale, which was more rapid on ECM of low rigidity [91]. Other cell interactions with ECM have been described on rapid times scale, such as microvascular endothelial cell spreading on fibronectin within minutes [150], fibroblast lamellipodia extension on fibronectin within seconds [150], and formation of MDA-MB-231 breast cancer cell protrusions and retractions on collagen within minutes [115]. Moreover, it is well known that cells modify ECM through biochemical means such as ECM deposition or MMP-dependent degradation, or through mechanical means like deforming, densifying, stiffening, and aligning ECM [106,107,108,109]. It is likely that the mechanical aspects of ECM serve as acute and dynamic mechanostressors for mammary tissue, and likely also operate in a dynamic reciprocal manner, but more work in normal breast epithelial cells and mammary tissue is required to firmly establish mechanism.

## 5. Mechanical Forces and Signaling in Breast Cancer

**Figure 3:**
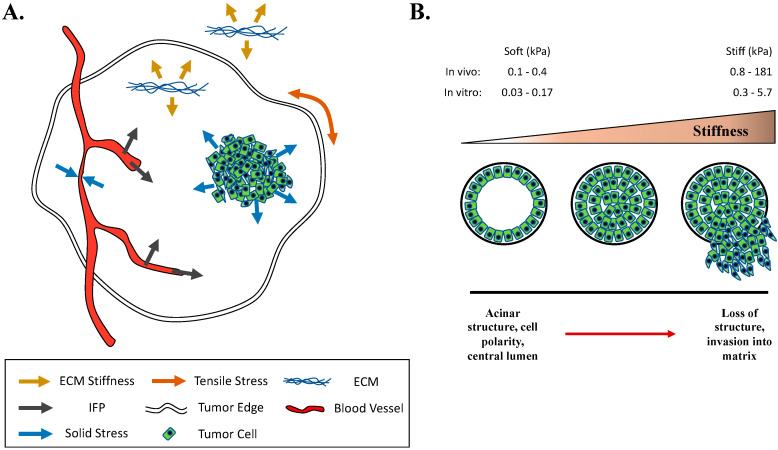
Mechanical signals in the tumor microenvironment. **A:** Pathologic mechanical signals present in the tumor microenvironment include stiffness, interstitial fluid pressure (IFP), solid stress, and tensile circumferential stress. **B:** Stiffness, a clinical risk factor for breast cancer, increases during tumor progression in vivo. In vitro studies show that stiffness can push normal mammary epithelial cells toward a malignant phenotype. As stiffness increases, the normal acinar structures formed by MCF10A cells become disrupted and cells become invasive into the extracellular matrix.

So far, we have discussed how mechanical stress can stimulate responses in non-tumorigenic breast epithelial cells and regulate mammary function, however, there is much more information on the presence and effects of mechanical stimuli in breast cancer (Figure 3A). Prior to tumor formation, alterations in the mechanical microenvironment of breast tissue are observed in patients at risk for developing cancer. For example, breast tissue density in vivo is a clinical risk factor for breast cancer [151,152,153], which is in part due to increased collagen, the major structural protein in the mammary gland, in patients [154], and may be independent of changes in glandular tissue [155]. Mechanistic insights come from in vitro studies, which show that increasing collagen concentration increases the stiffness of culture substrates and induces a FAK-dependent invasive phenotype in normal mammary epithelial cells [156]. Moreover, collagen density increases breast tumor formation and lung metastasis in vivo [157]. Considering breast density with other risk factors is a more complex issue; for example, excess total body fat (i.e., obesity) is also an established risk factor for breast cancer [158,159]. This could pose an interesting paradox by which low density areas of fat and high density areas of protein are equal risk factors [160], however, the specific role of excess fat in the breast and cancer risk has not firmly been established. Alternatively, aberrant mechanical signaling and high breast adipocyte content may work synergistically, since adipocytes are also mechanically sensitive [161]. Another clinical risk factor for breast cancer is tissue stiffness [162], which so far seems to be an independent risk factor from high breast density [163]. Indeed, breast carcinomas are characterized as being more stiff than glandular tissue [134]. This has led to new clinical technologies that show that stiffness evaluated by ultrasound-based sound touch elastography may aid breast cancer diagnosis [164] while stiffness measured by ultrasound-based shear wave elastography may provide prognostic value [165,166]. The effects and underlying mechanisms of stiffness on normal mammary tissue and tumors have been studied in greater detail and will be discussed further.

Once tumors have formed, alterations in the mechanical microenvironment of tumors are also observed, which includes changes in extracellular stiffness. Stiffness not only describes malignant lesions compared with non-malignant adjacent tissue in vivo, but also correlates with tumor progression. Ex vivo AFM measurements on fixed sections of human breast tissue and lesions showed that stiffness in normal tissue and non-invasive stroma was about 0.4 kPa, while the invasive fronts of invasive luminal ductal carcinoma (IDC) showed distributions ranging from 2–6 kPa (with some > 8 kPa) [167]. Using freshly excised mammary tumors from MMTV-PyMT mice, Lopez et al. report stiffening during tumor progression measured directly using AFM (normal mammary gland ducts = average of 0.4 kPa, 10-week minimally invasive tumors = average 1.2 kPa, 14-week highly invasive tumors = average 3 kPa) [168]. Other methods, such as the unconfined compression of mouse mammary glands and malignant tissues (via mechanical indenter attached to a force transducer), additionally support stiffness differences (non-malignant tissue = 0.167 ± 0.31 kPa vs. breast cancer = 4.049 ± 0.938 kPa) [118]. Furthermore, studies in mice report step-wise stiffening between normal (~0.1–0.2 kPa), premalignant (~0.2–0.6 kPa), and invasive cancer (~0.8–1.5 kPa), using independent methods (unconfined compression and shear rheology) [119]. High average stiffness measured in vivo by shear wave elastography range is seen in human lobular (181 ± 67 kPa) and ductal tumors (139 ± 56 kPa) and stiffness increases with tumor grade (benign lesions defined as < 50 kPa, Grade 1 = 88 ± 62 kPa, Grade 2 = 143 ± 55 kPa, Grade 3 = 147 ± 58 kPa) [166]. Stiffness measured by sound touch elastography showed high average stiffness in malignant lesions (40.85 kPa) over benign lesions (19.02 kPa) [164]. Supersonic shear imaging was able to distinguish stiffness differences between fat, dense tissue, benign lesions and malignant lesions (3, 45, <80, and >100 kPa, respectively) [169]. Interestingly, there is typically higher stiffness in the periphery or edges of the tumors (known as the “stiff rim”) [164,166,170]. Many other reports using ultrasound-based techniques also find stiffness in breast cancer [171,172,173,174,175].

Abundant experimental evidence supports stiffness as a mechanical modifier of breast cancer and provides mechanistic insight. One of the most striking demonstrations that mechanical signals such as stiffness play a role in the promotion of tumor formation and progression comes from in vitro experiments employing normal non-malignant mammary epithelial cells cultured in 2D and 3D environments of increasing stiffness (Figure 3B). Seminal work by the Weaver group using stiffness as an experimental model suggests that a disruption of cell tensional homeostasis (exogenous vs. endogenous forces) leads to malignant cell phenotypes in breast cancer. When non-malignant MCF10A cells are cultured in 3D matrices of increasing collagen concentration from 1–3.4 mg/mL, the stiffness of the 3D matrix increases from 0.170 kPa to 1.2 kPa [118]. In 3D culture with stiffnesses (0.170 kPa) closely matching that of normal mammary tissue (0.167 kPa), MCF10A cells form structured, growth arrested polarized acini with a central lumen, but MCF10A cells in increasingly stiff environments (up to 1.2 kPa), that more closely mimicked tumor stiffness (4.049 kPa), showed consistently disrupted spheroid organization [118]. Alternatively, extracellular stiffness was modulated using crosslinked polyacrylamide gels (0.15 kPa to 5 kPa) to control for potential confounding effects of large concentrations in collagen ligand, and stiffness similarly pushed MCF10A cells toward disrupted morphology and invasion through basement membrane (beginning at 1 kPa after 20 days in culture) [118]. Mechanistically, this stiffness-induced malignant phenotype was possible through the promotion of Rho-dependent cytoskeletal contractility and ERK, ultimately resulting in mature integrin-based focal adhesions, while blocking Rho or ERK reversed the phenotype. Other in vitro studies have subsequently bolstered the notion that stiffness alone can lead to a malignant phenotype in otherwise non-malignant mammary epithelial cells [16,176,177,178]. Moreover, the use of interpenetrating networks (IPN, of alginate and rBM (Matrigel) matrix), which allow for the tuning of extracellular stiffness in 3D cultures independent of the potentially confounding effects of polymer concentration, cell-adhesion-ligand density, and ECM architecture, show that stiffness alone disrupts normal MCF10A acinar formation, including rotational movement, growth arrest, central lumen, apicobasal polarization, and leads to local invasion after 11–19 days in culture [176]. However, both stiffness and ECM composition must ultimately be considered, since MCF10A cells cultured in pure rBM (Matrigel) matrix of increasing stiffness suppressed the malignant phenotype [176]. In addition to the disruption of tensional homeostasis [118], EMT activation through twist1 nuclear translocation may also underlie stiffness-mediated promotion of malignant phenotypes in non-tumorigenic MCF10A cells [16]. Translation of these ideas into therapeutics may be possible as targeting matrix stiffness in vivo through the pharmacologic inhibition of the ECM cross-linking enzyme lysyl oxidase with β-aminopropionitrile (BAPN) was able to reduce mammary tumor incidence and volume [119].

Another source of mechanical stress in the tumor microenvironment is an increased interstitial fluid pressure (IFP) compared with adjacent non-malignant tissue (sometimes also referred to as interstitial hypertension) (Figure 3A). High IFP was first recorded in the 1950s in rabbit testicular cancer [179], and was subsequently confirmed in other malignancies, including breast cancer (both rat [180] and human [181,182]). IFP is measured by hypodermic needles inserted directly into tumors and connected to pressure transducers for quantification, a technique that has also been adapted for recording in patients under anesthesia [181]. High IFP in breast cancer seems to be independent of tumor size [180,181], but correlated with tumor grade [181], however, one study reported opposite conclusions (correlations with size not grade) [182]. For mammary carcinoma, adjacent normal tissue average IFP is ~0 mmHg, while breast carcinoma IFP is 15 ± 9 mmHg [181], and a gradient of IFP exists where high IFP is uniform within tumors, but drops rapidly at the periphery (about 1 mm from the tumor surface) [183]. High IFP in breast tumors seems to be due to high vascular permeability [184] and the compression of blood vessels [185], and is driven by hydrostatic microvascular pressure (MVP), which has been confirmed through direct measurements and comparison of both MVP and IFP in mammary tumors [180]. The prognostic value of IFP has also been studied, at least in cervix cancer [186]. Modeling suggests that IFP results in fluid flow in vivo [187], which is supported by direct measurements of ~0.13–0.2 μm/sec flow velocity in breast tumors [188], or an average of 0.6 μm/sec in neoplastic tissue grown in rabbit ears [189]. Other experiments in glioma tumors measured an interstitial flow range of ~0.1–0.4 μm/sec [190]. To determine mechanisms by which IFP might affect breast cancer cells, researchers model in vitro IFP and/or fluid flow to show the acute and long-term responsiveness of these cells. For example, MDA-MB-231 cells in 2D culture respond within minutes to 2.5 and 10 dynes/cm^2^ of fluid flow shear stress with AMPK activation [141] and within 30 min to 1.8 dynes/cm^2^ with phosphorylation of FAK and reductions in acetylated tubulin [191]. To better model in vivo IFP, researchers apply hydrostatic pressure gradients across cells embedded in 3D matrices. A 60 kPa pressure gradient across a 2 mg/mL collagen gel containing MDA-MB-231 cells induced a 4.6 μm/s flow (∼1 Pa (1 pN/μm^2^) average stress), with a stress profile distinct from that of shear stress in 2D, and cells responded to 4 h of fluid flow with focal adhesion and cell protrusion formation in the direction of flow [142]. Similarly, a pressure gradient of ~1.2 mmHg across MDA-MB-231 cells embedded in collagen gels resulted in ~1 μm/s flow speed, which induced the expression of EMT proteins such as Snail and vimentin after six days, and promoted collective invasion [192]. Other 3D models have shown that proliferation and chemoresistance were increased via 5.4 dynes/cm^2^ of pulsatile fluid flow shear stress for 72 h in MDA-MB-231, MDA-MB-468, and MCF7 cells [193], that MDA-MB-435S cell invasion was enhanced by 18 h of 0.5 μm/s flow and further facilitated by associated fibroblasts [194], and that 24 h of 3.0 μm/s flow from 40 Pa pressure on MDA-MB-231 cells promoted directional migration [195]. The collective data illustrate a role for fluids as mechanical stressors in the tumor microenvironment in vivo and experimental models studying the effects of these mechanical stimuli show that they can enhance the malignant behaviors of cancer cells.

Solid stresses (Figure 3A), distinct from interstitial fluid pressure [180] and stiffness [133], are present in a variety of tumors, including breast tumors, and represent another form of mechanopathology [133,196]. Solid stress was presumed to be due to highly proliferating cells in tumors that impose on the surrounding ECM, as well as a reciprocal stress from ECM to the cell mass, but was not measured until it was modeled in vitro. By growing spheroids in a biologically inert matrix (agarose gels not broken down by cell degradation or permissive of cell migration) solid stress was calculated in mmHg using the size of the spheroids and mechanical properties of the gel, and was determined to fall within a range of 45–120 mmHg (6–16 kPa) [197]. This in vitro model was subsequently updated to reflect a measurement of 28 mmHg (3.7 kPa) [198]. Later work set out to measure in vivo solid stress using freshly excised human and animal tumors, including breast tumors, and reported ranges of 28–120 mmHg (3.7–16 kPa) for mouse tumors and 16.4–142.4 mmHg (2.2–19 kPa) for human tumors (for human breast tumors specifically, a range of = 10–19 kPa) [199]. These measurements were collected using a novel method for mathematically modeling deformations and displacements in tumors after making a single cut into the tumor (“based on the fundamental concept that tissues containing solid stress undergo deformation after release of physical confinement” [133]), where post-cut swelling (positive deformation) from the center of the tumor arose from compressive stress and tumor boundary retraction (negative deformation) was due to tensile circumferential stress [199]. More recently, researchers have compiled a range of techniques to measure compressive stress in both excised and in situ tumors [133,196]. This includes the planar-cut method (cut-based deformations, described above), the slicing method (cut-based deformations), and needle biopsy method (deformations in voided areas following core biopsy), where deformations are mapped using ultrasonography or optical coherence tomography, combined with mathematical modeling to quantify solid stress and elastic energy [133,196]. All three methods suggest that compressive stresses arise from the tumor center, while tensile stresses arise from the tumor periphery. Interestingly, solid stress increases with tumor size (measured in breast tumors) and, for some tumors, size-matched primary tumors have larger solid stress than metastatic tumors (shown in pancreatic tumors) [133]. Compressive stress from the high cell proliferation characteristic of tumors can compress blood and lymphatic vasculature [200], and this solid stress induced vessel compression contributes to hypoxia and acts as a barrier to drug delivery in breast tumors [201]. Furthermore, solid stress may contribute to low proliferation rates in tumor centers [202]. In addition to the in situ approaches, in vivo solid stress measured via noninvasive MR elastography is in development [203]. Studies modeling solid stress have shown that 2–4 weeks after in vivo induction of 1.2 kPa compressive stress (using implanted magnets) in colon tumors was able to activate the β-catenin pathway, independent of stiffness [204]. Other studies suggest that the neurologic dysfunction seen in patients with brain tumors is due to tumor mediated solid stress by showing that in vivo induction of solid stress resulted in neuronal damage, reductions in vascular perfusion, and impaired motor coordination [205]. Additionally, 16 h of in vitro 5.8 mmHg compressive stress (via weighted piston) was able to enhance MDA-MB-231 breast cancer cell invasive migration but suppressed MCF10A migration [206]. Overall, the data show that solid stress is a part of the mechanical microenvironment of breast tumors and that it can have biological effects on breast tumors and cancer cells. 

## 6. Conclusions

Mounting observational and experimental evidence supports the presence of a variety of mechanical stimuli in breast cancer that help promote malignancy. While there have been efforts to therapeutically target mechanical signals in vivo such as suppressing lysyl oxidase ECM crosslinking [119], this has not yet translated to changing clinical patient standard of care. Therefore, mechanical signaling and breast cancer may need a fresh perspective. One gap in knowledge is the mechanism by which non-tumorigenic breast epithelial cells and breast cancer cells respond and integrate acute mechanical stress. Of note, some mechanical stresses are more acute and dynamic than others. New ways of assessing the effects of mechanical stress in normal and malignant mammary epithelial cells may provide novel insights into the potential roles for the rapid mechano-chemical signaling that has already been demonstrated in many cell types. Indeed, mechanosensation and mechanotransduction mechanisms such as ion channel activation often operate on rapid timescales, and the rapid responsiveness of breast epithelial cells to mechanical stimuli, such as with Ca^2+^ signaling, has already been established. Especially when considering studies of breast cancer ECM stiffness that often rely on the long-term effects of mechanical signaling without sufficient temporal resolution, what role mechanosensors such as ion channels play in stiffness-inducing malignant phenotypes is of interest. The field of ECM stiffness and breast cancer biology may be shifting toward an earlier temporal resolution using models that employ or capture the dynamic stiffening of extracellular matrices. In an elegant study using time-lapse imaging of mammary MCF10A-Ras transformed acini, the authors demonstrated that acini mechanically remodel their surrounding substrates beginning at tens of minutes up to ~3 h, then they begin to spread by ~5 h, and finally show disruption of normal morphology by 10–20 h [207]. In a mechanism attributed to ROCK/myosin dependent contractility, the invasive phenotype could be reverted through mechanical isolation of the organoids [207]. One method using laser irradiation to stiffen 3D matrices on command resulted in invasive phenotypes in MCF10A cells 3 days post-stiffening [178], compared with that of 20 days in culture [118]. Similar results were independently reported several days post-stiffening [177]. It is also worth mentioning studies that test other methods for mechanical stimulation and their effects in mammary epithelial cells. In a clever system employed by Bissell, Fletcher and colleagues, T4–2 breast cancer cells that normally form disrupted acinar structures in 3D culture were phenotypically reversed into normal rotating acinar structures, with a central lumen in a process termed mechanical reversion [208]. Interestingly, this was possible only when compression was applied shortly after seeding cells into 3D matrices. In other studies using a flexible silicone culture system, non-tumorigenic mammary HC11 cells subjected to mechanical stretch responded with increased c-fos protein expression (after 60 min of mechanical strain) and ERK1/2 phosphorylation (after 5 min of mechanical strain) [131]. Finally, one method sets the stage for monitoring both extracellular stiffness and dynamic mechanical stress using cells plated on substrates of different stiffness coupled to transient substrate stretch or “tugging” via magnetic manipulation [209]. Following similar approaches, it may be possible to experimentally define novel signaling mechanisms by which breast cancer cells integrate acute mechanical cues, and set the foundation for further drug discovery.

In conclusion, aberrant mechanotransduction is characteristic of the tumor microenvironment which promotes a malignant phenotype in breast epithelial cells and exacerbates tumor cell behavior. Further insight into the mechanisms that underlie these effects could provide new molecular targets to reduce cancer progression. Indeed, there have been new efforts to discover novel mechanosensors which may provide new details. For example, the g-protein coupled receptor (GPCR) GPR68 has recently been identified as a novel sensor of fluid flow necessary for vascular physiology [210], and may be an emerging therapeutic target in cancer [211]. Mechanically sensitive adhesion GPCRs [212] are another set of potential targets, such as GPR116 which can promote actomyosin contractility and breast cancer metastasis [213]. Furthermore, immune cell mechanosensing is a rapidly evolving field [214] that may represent an indirect mechanism in breast cancer progression, as it could also be modified by enhanced mechanosignaling in the tumor microenvironment. At first, the evidence suggests a potential mechanism by which mechanosignaling promotes immune activation against cancer. T cell receptors can be activated directly by force [11,215] and T cell cytotoxicity increased by force [216]. Moreover, T cell [217] and B cell [218] activation can become enhanced on stiff substrates. However, pro-tumorigenic macrophage infiltration was observed specifically at breast cancer tumor invasive edges and correlated with edge stiffness, collagen linearization and TGFβ signaling [167], a mechanism further exacerbated in the more aggressive HER2 and basal-like cancers. Therefore, a stiffening tumor microenvironment could promote rather than inhibit tumor progression via immune cells. Defining specific molecular therapeutic targets in mechanotransduction pathways for cancer treatment, such as targeting novel mechanosensors, may help dampen aberrant tumor mechanosignaling in a new way. In contrast, current microtubule-targeting therapies commonly used to reduce tumor growth result in microtubule destabilization (Vinca alkaloids, Halichodrins) or microtubule hyperstabilization (Taxanes, Epothilones) [219,220]. Microtubules are known translators of mechanical signals such as tension within the cell and are involved in mechanosensing mechanisms [3,122,221]. This non-specific approach likely influences the mechanosensitivity of cells throughout the body, but could also influence the mechanosensitivity of cancer cells that survive the treatment [220]. Careful examination of changes in the tumor mechanical microenvironment, as well as tumor cell mechanosignaling, as a result of these current standards of care therapies, is needed.

## References

[1] Gillespie P.G., Muller U. (2009). Mechanotransduction by hair cells: Models, molecules, and mechanisms. Cell.

[2] Corey D.P., Hudspeth A.J. (1983). Kinetics of the receptor current in bullfrog saccular hair cells. J. Neurosci..

[3] Lyons J.S., Joca H.C., Law R.A., Williams K., Kerr J.P., Shi G., Khairallah R.J., Martin S.S., Konstantopoulos K., Ward C.W. (2017). Microtubules tune mechanotransduction through NOX2 and TRPV4 to decrease sclerostin abundance in osteocytes. Sci. Signal..

[4] Prosser B.L., Ward C., Lederer W. (2011). X-ROS signaling: Rapid mechano-chemo transduction in heart. Science.

[5] Alenghat F.J., Nauli S.M., Kolb R., Zhou J., Ingber D.E. (2004). Global cytoskeletal control of mechanotransduction in kidney epithelial cells. Exp. Cell Res..

[6] Nauli S.M., Alenghat F.J., Luo Y., Williams E., Vassilev P., Li X., Elia A.E.H., Lu W., Brown E.M., Quinn S. (2003). Polycystins 1 and 2 mediate mechanosensation in the primary cilium of kidney cells. Nat. Genet..

[7] Praetorius H.A., Spring K. (2001). Bending the MDCK cell primary cilium increases intracellular calcium. J. Membr. Biol..

[8] Qiu Y., Brown A.C., Myers D.R., Sakurai Y., Mannino R.G., Tran R., Ahn B., Hardy E.T., Kee M.F., Kumar S. (2014). Platelet mechanosensing of substrate stiffness during clot formation mediates adhesion, spreading, and activation. Proc. Natl. Acad. Sci. USA.

[9] Wang K.-C., Yeh Y.-T., Nguyen P., Limqueco E., Lopez J., Thorossian S., Guan K.-L., Li Y.-S.J., Chien S. (2016). Flow-dependent YAP/TAZ activities regulate endothelial phenotypes and atherosclerosis. Proc. Natl. Acad. Sci. USA.

[10] Desai S.S., Tung J.C., Zhou V.X., Grenert J.P., Malato Y., Rezvani M., Espanol-Suner R., Willenbring H., Weaver V.M., Chang T.T. (2016). Physiological ranges of matrix rigidity modulate primary mouse hepatocyte function in part through hepatocyte nuclear factor 4 alpha. Hepatology.

[11] Kim S.T., Takeuchi K., Sun Z.Y., Touma M., Castro C.E., Fahmy A., Lang M.J., Wagner G., Reinherz E.L. (2009). The alphabeta T cell receptor is an anisotropic mechanosensor. J. Biol. Chem..

[12] Wang J.H. (2020). T cell receptors, mechanosensors, catch bonds and immunotherapy. Prog. Biophys. Mol. Biol..

[13] Shoham N., Gottlieb R., Sharabani-Yosef O., Zaretsky U., Benayahu D., Gefen A. (2012). Static mechanical stretching accelerates lipid production in 3T3-L1 adipocytes by activating the MEK signaling pathway. Am. J. Physiol. Physiol..

[14] Erickson G.R., Alexopoulos L.G., Guilak F. (2001). Hyper-osmotic stress induces volume change and calcium transients in chondrocytes by transmembrane, phospholipid, and G-protein pathways. J. Biomech..

[15] Park J.S., Burckhardt C.J., Lazcano R., Solis L.M., Isogai T., Li L., Chen C.S., Gao B., Minna J.D., Bachoo R. (2020). Mechanical regulation of glycolysis via cytoskeleton architecture. Nature.

[16] Wei S.C., Fattet L., Tsai J.H., Guo Y., Pai V.H., Majeski H.E., Chen A.C., Sah R.L., Taylor S.S., Engler A.J. (2015). Matrix stiffness drives epithelial–mesenchymal transition and tumour metastasis through a TWIST1–G3BP2 mechanotransduction pathway. Nat. Cell Biol..

[17] Gudipaty S.A., Lindblom J., Loftus P.D., Redd M.J., Edes K., Davey C.F., Krishnegowda V., Rosenblatt J. (2017). Mechanical stretch triggers rapid epithelial cell division through Piezo1. Nature.

[18] Gasparski A., Ozarkar S., Beningo K.A. (2017). Transient mechanical strain promotes the maturation of invadopodia and enhances cancer cell invasion in vitro. J. Cell Sci..

[19] Li Y., Ge C., Long J.P., Begun D.L., Rodríguez J.A., Goldstein S.A., Franceschi R.T. (2012). Biomechanical stimulation of osteoblast gene expression requires phosphorylation of the RUNX2 transcription factor. J. Bone Miner. Res..

[20] Marshall K.L., Lumpkin E.A. (2012). The molecular basis of mechanosensory transduction. Adv. Exp. Med. Biol..

[21] Anishkin A., Sukharev S. (2004). Water dynamics and dewetting transitions in the small mechanosensitive channel MscS. Biophys. J..

[22] Chiang C.-S., Anishkin A., Sukharev S. (2004). Gating of the large mechanosensitive channel in situ: Estimation of the spatial scale of the transition from channel population responses. Biophys. J..

[23] Cox C.D., Bae C., Ziegler L., Hartley S., Nikolova-Krstevski V., Rohde P.R., Ng C.-A., Sachs F., Gottlieb P., Martinac B. (2016). Removal of the mechanoprotective influence of the cytoskeleton reveals PIEZO1 is gated by bilayer tension. Nat. Commun..

[24] Chang G., Spencer R.H., Lee A.T., Barclay M.T., Rees D.C. (1998). Structure of the MscL homolog from mycobacterium tuberculosis: A gated mechanosensitive ion channel. Science.

[25] Betanzos M., Chiang C.S., Guy H.R., Sukharev S. (2002). A large iris-like expansion of a mechanosensitive channel protein induced by membrane tension. Nat. Struct. Biol..

[26] Perozo E., Cortes D.M., Sompornpisut P., Kloda A., Martinac B. (2002). Open channel structure of MscL and the gating mechanism of mechanosensitive channels. Nature.

[27] Corry B., Rigby P., Liu Z.W., Martinac B. (2005). Conformational changes involved in MscL channel gating measured using FRET spectroscopy. Biophys. J..

[28] Zhao Q., Zhou H., Chi S., Wang Y., Wang J., Geng J., Wu K., Liu W., Zhang T., Dong M.Q. (2018). Structure and mechanogating mechanism of the Piezo1 channel. Nature.

[29] Saotome K., Murthy S.E., Kefauver J.M., Whitwam T., Patapoutian A., Ward A.B. (2017). Structure of the mechanically activated ion channel Piezo1. Nature.

[30] Wang L., Zhou H., Zhang M., Liu W., Deng T., Zhao Q., Li Y., Lei J., Li X., Xiao B. (2019). Structure and mechanogating of the mammalian tactile channel PIEZO2. Nature.

[31] Wu J., Young M., Lewis A.H., Martfeld A.N., Kalmeta B., Grandl J. (2017). Inactivation of mechanically activated piezo1 ion channels is determined by the c-terminal extracellular domain and the inner pore helix. Cell Rep..

[32] Lewis A.H., Grandl J. (2015). Mechanical sensitivity of Piezo1 ion channels can be tuned by cellular membrane tension. eLife.

[33] Gnanasambandam R., Bae C., Gottlieb P., Sachs F. (2015). Ionic selectivity and permeation properties of human PIEZO1 channels. PLoS ONE.

[34] Zheng J. (2013). Molecular mechanism of TRP channels. Compr. Physiol..

[35] Clapham D.E., Runnels L.W., Strubing C. (2001). The TRP ion channel family. Nat. Rev. Neurosci..

[36] Nikolaev Y.A., Cox C.D., Ridone P., Rohde P.R., Cordero-Morales J.F., Vásquez V., Laver D.R., Martinac B. (2019). Mammalian TRP ion channels are insensitive to membrane stretch. J. Cell Sci..

[37] Christensen A.P., Corey D.P. (2007). TRP channels in mechanosensation: Direct or indirect activation?. Nat. Rev. Neurosci..

[38] Liedtke W., Choe Y., Marti-Renom M.A., Bell A.M., Denis C.S., Sali A., Hudspeth A.J., Friedman J.M., Heller S. (2000). Vanilloid receptor-related osmotically activated channel (VR-OAC), a candidate vertebrate osmoreceptor. Cell.

[39] Wu L., Gao X., Brown R.C., Heller S., O’Neil R.G. (2007). Dual role of the TRPV4 channel as a sensor of flow and osmolality in renal epithelial cells. Am. J. Physiol. Renal Physiol..

[40] Loukin S.H., Zhou X., Su Z., Saimi Y., Kung C. (2010). Wild-type and Brachyolmia-causing mutant TRPV4 channels respond directly to stretch force. J. Biol. Chem..

[41] Morita H., Honda A., Inoue R., Ito Y., Abe K., Nelson M.T., Brayden J.E. (2007). Membrane stretch-induced activation of a TRPM4-like nonselective cation channel in cerebral artery myocytes. J. Pharmacol. Sci..

[42] Numata T., Shimizu T., Okada Y. (2007). TRPM7 is a stretch- and swelling-activated cation channel involved in volume regulation in human epithelial cells. Am. J. Physiol. Physiol..

[43] Maroto R., Raso A., Wood T.G., Kurosky A., Martinac B., Hamill O.P. (2005). TRPC1 forms the stretch-activated cation channel in vertebrate cells. Nat. Cell Biol..

[44] Spassova M.A., Hewavitharana T., Xu W., Soboloff J., Gill D.L. (2006). A common mechanism underlies stretch activation and receptor activation of TRPC6 channels. Proc. Natl. Acad. Sci. USA.

[45] Zhang W., Cheng L.E., Kittelmann M., Li J., Petkovic M., Cheng T., Jin P., Guo Z., Göpfert M.C., Jan L.Y. (2015). Ankyrin repeats convey force to gate the NOMPC mechanotransduction channel. Cell.

[46] Matthews B.D., Thodeti C.K., Tytell J.D., Mammoto A., Overby D.R., Ingber D.E. (2010). Ultra-rapid activation of TRPV4 ion channels by mechanical forces applied to cell surface beta1 integrins. Integr. Biol. (Camb.).

[47] Brohawn S.G., Su Z., MacKinnon R. (2014). Mechanosensitivity is mediated directly by the lipid membrane in TRAAK and TREK1 K+ channels. Proc. Natl. Acad. Sci. USA.

[48] Brohawn S.G., Campbell E.B., MacKinnon R. (2014). Physical mechanism for gating and mechanosensitivity of the human TRAAK K+ channel. Nature.

[49] Sun Z., Costell M., Fässler R. (2019). Integrin activation by talin, kindlin and mechanical forces. Nat. Cell Biol..

[50] Sun Z., Guo S.S., Fässler R. (2016). Integrin-mediated mechanotransduction. J. Cell Biol..

[51] Kechagia J.Z., Ivaska J., Roca-Cusachs P. (2019). Integrins as biomechanical sensors of the microenvironment. Nat. Rev. Mol. Cell Biol..

[52] Lecuit T., Yap A.S. (2015). E-cadherin junctions as active mechanical integrators in tissue dynamics. Nat. Cell Biol..

[53] Pannekoek W.-J., De Rooij J., Gloerich M. (2019). Force transduction by cadherin adhesions in morphogenesis. F1000Research.

[54] Kim J., Lee J., Jang J., Ye F., Hong S.J., Petrich B.G., Ulmer T.S., Kim C. (2020). Topological adaptation of transmembrane domains to the force-modulated lipid bilayer is a basis of sensing mechanical force. Curr. Biol..

[55] Zhu J., Luo B.-H., Xiao T.S., Zhang C., Nishida N., Springer T.A. (2008). Structure of a complete integrin ectodomain in a physiologic resting state and activation and deactivation by applied forces. Mol. Cell.

[56] Chen Y., Lee H., Tong H., Schwartz M., Zhu C. (2017). Force regulated conformational change of integrin alphaVbeta3. Matrix Biol..

[57] Li J., Springer T.A. (2017). Integrin extension enables ultrasensitive regulation by cytoskeletal force. Proc. Natl. Acad. Sci. USA.

[58] Kong F., Garcia A.J., Mould A.P., Humphries M.J., Zhu C. (2009). Demonstration of catch bonds between an integrin and its ligand. J. Cell Biol..

[59] Yao M., Goult B.T., Klapholz B., Hu X., Toseland C.P., Guo Y., Cong P., Sheetz M.P., Yan J. (2016). The mechanical response of talin. Nat. Commun..

[60] Del Rio A., Perez-Jimenez R., Liu R., Roca-Cusachs P., Fernandez J.M., Sheetz M.P. (2009). Stretching single talin rod molecules activates vinculin binding. Science.

[61] Yao M., Goult B.T., Chen H., Cong P., Sheetz M.P., Yan J. (2014). Mechanical activation of vinculin binding to talin locks talin in an unfolded conformation. Sci. Rep..

[62] Elosegui-Artola A., Oria R., Chen Y., Kosmalska A., Pérez-González C., Castro N., Zhu C., Trepat X., Roca-Cusachs P. (2016). Mechanical regulation of a molecular clutch defines force transmission and transduction in response to matrix rigidity. Nature.

[63] Paszek M.J., Dufort C.C., Rossier O., Bainer R., Mouw J.K., Godula K., Hudak J.E., Lakins J.N., Wijekoon A.C., Cassereau L. (2014). The cancer glycocalyx mechanically primes integrin-mediated growth and survival. Nature.

[64] Paszek M.J., Boettiger D., Weaver V.M., Hammer D.A. (2009). Integrin clustering is driven by mechanical resistance from the glycocalyx and the substrate. PLoS Comput. Biol..

[65] Cavalcanti-Adam E.A., Volberg T., Micoulet A., Kessler H., Geiger B., Spatz J.P. (2007). Cell spreading and focal adhesion dynamics are regulated by spacing of integrin ligands. Biophys. J..

[66] Murrell M., Oakes P.W., Lenz M., Gardel M.L. (2015). Forcing cells into shape: The mechanics of actomyosin contractility. Nat. Rev. Mol. Cell Biol..

[67] Pandya P., Orgaz J.L., Sanz-Moreno V. (2017). Actomyosin contractility and collective migration: May the force be with you. Curr. Opin. Cell Biol..

[68] Tharp K.M., Kang M.S., Timblin G., Dempersmier J., Dempsey G.E., Zushin P.-J.H., Benavides J., Choi C., Li C.X., Jha A.K. (2018). Actomyosin-mediated tension orchestrates uncoupled respiration in adipose tissues. Cell Metab..

[69] Takaki T., Montagner M., Serres M.P., Le Berre M., Russell M.R., Collinson L.M., Szuhai K., Howell M., Boulton S.J., Sahai E. (2017). Actomyosin drives cancer cell nuclear dysmorphia and threatens genome stability. Nat. Commun..

[70] Samuel M.S., Lopez J.I., McGhee E.J., Croft D.R., Strachan D., Timpson P., Munro J., Schroder E., Zhou J., Brunton V.G. (2011). Actomyosin-mediated cellular tension drives increased tissue stiffness and beta-catenin activation to induce epidermal hyperplasia and tumor growth. Cancer Cell.

[71] Poincloux R., Collin O., Lizárraga F., Romao M., Debray M., Piel M., Chavrier P. (2011). Contractility of the cell rear drives invasion of breast tumor cells in 3D Matrigel. Proc. Natl. Acad. Sci. USA.

[72] Hamidi H., Ivaska J. (2018). Every step of the way: Integrins in cancer progression and metastasis. Nat. Rev. Cancer.

[73] Yoshimura K., Meckel K.F., Laird L.S., Chia C.Y., Park J.J., Olino K.L., Tsunedomi R., Harada T., Iizuka N., Hazama S. (2009). Integrin alpha2 mediates selective metastasis to the liver. Cancer Res..

[74] Zhou B., Gibson-Corley K.N., Herndon M.E., Sun Y., Gustafson-Wagner E., Teoh-Fitzgerald M., Domann F.E., Henry M.D., Stipp C.S. (2014). Integrin alpha3beta1 can function to promote spontaneous metastasis and lung colonization of invasive breast carcinoma. Mol. Cancer Res..

[75] Wang L., Song L., Li J., Wang Y., Yang C., Kou X., Xiao B., Zhang W., Li L., Liu S. (2019). Bone sialoprotein-alphavbeta3 integrin axis promotes breast cancer metastasis to the bone. Cancer Sci..

[76] Rakshit S., Zhang Y., Manibog K., Shafraz O., Sivasankar S. (2012). Ideal, catch, and slip bonds in cadherin adhesion. Proc. Natl. Acad. Sci. USA.

[77] Manibog K., Li H., Rakshit S., Sivasankar S. (2014). Resolving the molecular mechanism of cadherin catch bond formation. Nat. Commun..

[78] Buckley C.D., Tan J., Anderson K.L., Hanein R., Volkmann N., Weis W.I., Nelson W.J., Dunn A.R. (2014). The minimal cadherin-catenin complex binds to actin filaments under force. Science.

[79] Ishiyama N., Sarpal R., Wood M.N., Barrick S.K., Nishikawa T., Hayashi H., Kobb A.B., Flozak A.S., Yemelyanov A., Fernandez-Gonzalez R. (2018). Force-dependent allostery of the α-catenin actin-binding domain controls adherens junction dynamics and functions. Nat. Commun..

[80] Yonemura S., Wada Y., Watanabe T., Nagafuchi A., Shibata M. (2010). α-Catenin as a tension transducer that induces adherens junction development. Nature.

[81] Yao M., Qiu W., Liu R., Efremov A.K., Cong P., Seddiki R., Payre M., Lim C.T., Ladoux B., Mege R.M. (2014). Force-dependent conformational switch of alpha-catenin controls vinculin binding. Nat. Commun..

[82] Huang D.L., Bax N.A., Buckley C.D., I Weis W., Dunn A.R. (2017). Vinculin forms a directionally asymmetric catch bond with F-actin. Science.

[83] Thomas W.A., Boscher C., Chu Y.S., Cuvelier D., Martinez-Rico C., Seddiki R., Heysch J., Ladoux B., Thiery J.P., Mege R.M. (2013). Alpha-Catenin and vinculin cooperate to promote high E-cadherin-based adhesion strength. J. Biol. Chem..

[84] Maruthamuthu V., Sabass B., Schwarz U.S., Gardel M.L. (2011). Cell-ECM traction force modulates endogenous tension at cell–cell contacts. Proc. Natl. Acad. Sci. USA.

[85] Price A., Cost A.-L., Ungewiß H., Waschke J., Dunn A.R., Grashoff C. (2018). Mechanical loading of desmosomes depends on the magnitude and orientation of external stress. Nat. Commun..

[86] Berridge M.J., Bootman M., Roderick H.L. (2003). Calcium signalling: Dynamics, homeostasis and remodelling. Nat. Rev. Mol. Cell Biol..

[87] Li J., Hou B., Tumova S., Muraki K., Bruns A., Ludlow M.J., Sedo A., Hyman A., McKeown L., Young R.S. (2014). Piezo1 integration of vascular architecture with physiological force. Nature.

[88] Mousawi F., Peng H., Li J., Ponnambalam S., Roger S., Zhao H., Yang X., Jiang L.-H., Sreenivasan P. (2020). Chemical activation of the Piezo1 channel drives mesenchymal stem cell migration via inducing ATP release and activation of P2 receptor purinergic signaling. Stem Cells.

[89] Pardo-Pastor C., Rubio-Moscardó F., Vogel-González M., Serra S.A., Afthinos A., Mrkonjic S., Destaing O., Abenza J.F., Fernández-Fernández J.M., Trepat X. (2018). Piezo2 channel regulates RhoA and actin cytoskeleton to promote cell mechanobiological responses. Proc. Natl. Acad. Sci. USA.

[90] Han Y., Liu C., Zhang N., Men H., Huo L., Geng Q., Wang S., Gao Y., Zhang W., Zhang Y. (2019). Mechanosensitive ion channel Piezo1 promotes prostate cancer development through the activation of the Akt/mTOR pathway and acceleration of cell cycle. Int. J. Oncol..

[91] Plotnikov S.V., Pasapera A.M., Sabass B., Waterman C.M. (2012). Force fluctuations within focal adhesions mediate ECM-rigidity sensing to guide directed cell migration. Cell.

[92] Guilluy C., Swaminathan V., Garcia-Mata R., O’Brien E.T., Superfine R., Burridge K. (2011). The Rho GEFs LARG and GEF-H1 regulate the mechanical response to force on integrins. Nat. Cell Biol..

[93] Pawlowski R., Rajakyla E.K., Vartiainen M.K., Treisman R. (2010). An actin-regulated importin alpha/beta-dependent extended bipartite NLS directs nuclear import of MRTF-A. EMBO J..

[94] Plessner M., Melak M., Chinchilla P., Baarlink C., Grosse R. (2015). Nuclear F-actin formation and reorganization upon cell spreading. J. Biol. Chem..

[95] Wang N., Tytell J.D., Ingber D.E. (2009). Mechanotransduction at a distance: Mechanically coupling the extracellular matrix with the nucleus. Nat. Rev. Mol. Cell Biol..

[96] Isermann P., Lammerding J. (2013). Nuclear mechanics and mechanotransduction in health and disease. Curr. Biol..

[97] Ito S., Okuda S., Abe M., Fujimoto M., Onuki T., Nishimura T., Takeichi M. (2017). Induced cortical tension restores functional junctions in adhesion-defective carcinoma cells. Nat. Commun..

[98] De Pascalis C., Etienne-Manneville S. (2017). Single and collective cell migration: The mechanics of adhesions. Mol. Biol. Cell.

[99] Ladoux B., Mege R.M. (2017). Mechanobiology of collective cell behaviours. Nat. Rev. Mol. Cell Biol..

[100] Thorne J., Segal T.R., Chang S., Jorge S., Segars J.H., Leppert P. (2015). Dynamic reciprocity between cells and their microenvironment in reproduction. Biol. Reprod..

[101] Bissell M.J., Hall H., Parry G. (1982). How does the extracellular matrix direct gene expression?. J. Theor. Biol..

[102] Jorge S., Chang S., Barzilai J.J., Leppert P., Segars J. (2014). Mechanical signaling in reproductive tissues: Mechanisms and importance. Reprod. Sci..

[103] Xu R., Boudreau A., Bissell M.J. (2009). Tissue architecture and function: Dynamic reciprocity via extra- and intra-cellular matrices. Cancer Metastasis Rev..

[104] Paszek M.J., Weaver V.M. (2004). The tension mounts: Mechanics meets morphogenesis and malignancy. J. Mammary Gland Biol. Neoplasia.

[105] Van Helvert S., Storm C., Friedl P. (2017). Mechanoreciprocity in cell migration. Nat. Cell Biol..

[106] Hall M.S., Alisafaei F., Ban E., Feng X., Hui C.-Y., Shenoy V.B., Wu M. (2016). Fibrous nonlinear elasticity enables positive mechanical feedback between cells and ECMs. Proc. Natl. Acad. Sci. USA.

[107] van Helvert S., Friedl P. (2016). Strain stiffening of fibrillar collagen during individual and collective cell migration identified by AFM nanoindentation. ACS Appl. Mater. Interfaces.

[108] Gjorevski N., Piotrowski A.S., Varner V.D., Nelson C.M. (2015). Dynamic tensile forces drive collective cell migration through three-dimensional extracellular matrices. Sci. Rep..

[109] Kim J., Feng J., Jones C.A.R., Mao X., Sander L.M., Levine H., Sun B. (2017). Stress-induced plasticity of dynamic collagen networks. Nat. Commun..

[110] Solon J., Levental I., Sengupta K., Georges P.C., Janmey P.A. (2007). Fibroblast adaptation and stiffness matching to soft elastic substrates. Biophys. J..

[111] Lo C.-M., Wang H.-B., Dembo M., Wang Y.-L. (2000). Cell movement is guided by the rigidity of the substrate. Biophys. J..

[112] Vincent L.G., Choi Y.S., Alonso-Latorre B., Del Alamo J.C., Engler A.J. (2013). Mesenchymal stem cell durotaxis depends on substrate stiffness gradient strength. Biotechnol. J..

[113] Sunyer R., Conte V., Escribano J., Elosegui-Artola A., Labernadie A., Valon L., Navajas D., Garcia-Aznar J.M., Munoz J.J., Roca-Cusachs P. (2016). Collective cell durotaxis emerges from long-range intercellular force transmission. Science.

[114] Tamariz E., Grinnell F. (2002). Modulation of fibroblast morphology and adhesion during collagen matrix remodeling. Mol. Biol. Cell.

[115] Carey S.P., Goldblatt Z.E., Martin K.E., Romero B., Williams R.M., Reinhart-King C.A. (2016). Local extracellular matrix alignment directs cellular protrusion dynamics and migration through Rac1 and FAK. Integr. Biol. (Camb.).

[116] Wolf K., Te Lindert M., Krause M., Alexander S., Te Riet J., Willis A.L., Hoffman R.M., Figdor C.G., Weiss S.J., Friedl P. (2013). Physical limits of cell migration: Control by ECM space and nuclear deformation and tuning by proteolysis and traction force. J. Cell Biol..

[117] Wolf K., Wu Y.I., Liu Y., Geiger J., Tam E., Overall C., Stack M.S., Friedl P. (2007). Multi-step pericellular proteolysis controls the transition from individual to collective cancer cell invasion. Nat. Cell Biol..

[118] Paszek M.J., Zahir N., Johnson K.R., Lakins J.N., Rozenberg G.I., Gefen A., Reinhart-King C.A., Margulies S.S., Dembo M., Boettiger D. (2005). Tensional homeostasis and the malignant phenotype. Cancer Cell.

[119] Levental K.R., Yu H., Kass L., Lakins J.N., Egeblad M., Erler J.T., Fong S.F., Csiszar K., Giaccia A., Weninger W. (2009). Matrix crosslinking forces tumor progression by enhancing integrin signaling. Cell.

[120] Toh R., Shinohara M., Takaya T., Yamashita T., Masuda S., Kawashima S., Yokoyama M., Yagi N. (2006). An X-Ray diffraction study on mouse cardiac cross-bridge function in vivo: Effects of adrenergic {beta}-stimulation. Biophys. J..

[121] Prosser B.L., Khairallah R.J., Ziman A.P., Ward C., Lederer W. (2012). X-ROS signaling in the heart and skeletal muscle: Stretch-dependent local ROS regulates [Ca^2+^]i. J. Mol. Cell. Cardiol..

[122] Kerr J.P., Robison P., Shi G., Bogush A.I., Kempema A.M., Hexum J.K., Becerra N., Harki D.A., Martin S.S., Raiteri R. (2015). Detyrosinated microtubules modulate mechanotransduction in heart and skeletal muscle. Nat. Commun..

[123] Gardinier J.D., Townend C.W., Jen K.-P., Wu Q., Duncan R.L., Wang L. (2010). In situ permeability measurement of the mammalian lacunar–canalicular system. Bone.

[124] Price C., Zhou X., Li W., Wang L. (2010). Real-time measurement of solute transport within the lacunar-canalicular system of mechanically loaded bone: Direct evidence for load-induced fluid flow. J. Bone Miner. Res..

[125] Lyons J.S., Iyer S.R., Lovering R.M., Ward C.W., Stains J.P. (2016). Novel multi-functional fluid flow device for studying cellular mechanotransduction. J. Biomech..

[126] Mammoto T., Ingber D.E. (2010). Mechanical control of tissue and organ development. Development.

[127] Mammoto A., Mammoto T., Ingber D.E. (2012). Mechanosensitive mechanisms in transcriptional regulation. J. Cell Sci..

[128] Alcaraz J., Nelson C.M., Bissell M.J. (2004). Biomechanical approaches for studying integration of tissue structure and function in mammary epithelia. J. Mammary Gland. Biol. Neoplasia.

[129] Schedin P., Keely P.J. (2010). Mammary gland ECM remodeling, stiffness, and mechanosignaling in normal development and tumor progression. Cold Spring Harb. Perspect. Biol..

[130] Marti A., Feng Z., Altermatt H.J., Jaggi R. (1997). Milk accumulation triggers apoptosis of mammary epithelial cells. Eur. J. Cell Biol..

[131] Quaglino A., Salierno M., Pellegrotti J.V., Rubinstein N., Kordon E.C. (2009). Mechanical strain induces involution-associated events in mammary epithelial cells. BMC Cell Biol..

[132] Macias H., Hinck L. (2012). Mammary gland development. Wiley Interdiscip. Rev. Dev. Biol..

[133] Nia H.T., Liu H., Seano G., Datta M., Jones D., Rahbari N., Incio J., Chauhan V., Jung K., Martin J.D. (2016). Solid stress and elastic energy as measures of tumour mechanopathology. Nat. Biomed. Eng..

[134] Krouskop T.A., Wheeler T.M., Kallel F., Garra B.S., Hall T. (1998). Elastic moduli of breast and prostate tissues under compression. Ultrason Imaging.

[135] Gefen A., Dilmoney B. (2007). Mechanics of the normal woman’s breast. Technol. Health Care.

[136] Enomoto K.-I., Furuya K., Maeno T., Edwards C., Oka T. (1987). Mechanically induced electrical responses in murine mammary epithelial cells in primary culture. FEBS Lett..

[137] Furuya K., Enomoto K.-I. (1990). Real-time imaging of intracellular calcium change with simultaneous single channel recording in mammary epithelial cells. Brain Res. Bull..

[138] Enomoto K., Furuya K., Yamagishi S., Maeno T. (1992). Mechanically induced electrical and intracellular calcium responses in normal and cancerous mammary cells. Cell Calcium.

[139] Furuya K., Enomoto K.-I., Yamagishi S. (1993). Spontaneous calcium oscillations and mechanically and chemically induced calcium responses in mammary epithelial cells. Pflug. Arch..

[140] Enomoto K.-I., Furuya K., Yamagishi S., Oka T., Maeno T. (1994). The increase in the intracellular Ca2+ concentration induced by mechanical stimulation is propagated via release of pyrophosphorylated nucleotides in mammary epithelial cells. Pflug. Arch..

[141] Guo Y., Steele H.E., Li B.-Y., Na S. (2020). Fluid flow-induced activation of subcellular AMPK and its interaction with FAK and Src. Arch. Biochem. Biophys..

[142] Polacheck W., German A.E., Mammoto A., Ingber N.E., Kamm R.D. (2014). Mechanotransduction of fluid stresses governs 3D cell migration. Proc. Natl. Acad. Sci. USA.

[143] Rajakyla E.K., Lehtimaki J.I., Acheva A., Schaible N., Lappalainen P., Krishnan R., Tojkander S. (2020). Assembly of peripheral actomyosin bundles in epithelial cells is dependent on the CaMKK2/AMPK pathway. Cell Rep..

[144] Birchmeier C., Birchmeier W., Brand-Saberi B. (1996). Epithelial-mesenchymal transitions in cancer progression. Acta Anat..

[145] Wozniak M.A., Desai R., Solski P.A., Der C.J., Keely P.J. (2003). ROCK-generated contractility regulates breast epithelial cell differentiation in response to the physical properties of a three-dimensional collagen matrix. J. Cell Biol..

[146] Emerman J.T., Pitelka D.R. (1977). Maintenance and induction of morphological differentiation in dissociated mammary epithelium on floating collagen membranes. Vitro.

[147] Lee E.Y., Parry G., Bissell M.J. (1984). Modulation of secreted proteins of mouse mammary epithelial cells by the collagenous substrata. J. Cell Biol..

[148] Parry G., Lee E., Farson D., Koval M., Bissell M. (1985). Collagenous substrata regulate the nature and distribution of glycosaminoglycans produced by differentiated cultures of mouse mammary epithelial cells. Exp. Cell Res..

[149] Gehler S., Baldassarre M., Lad Y., Leight J.L., Wozniak M.A., Riching K.M., Eliceiri K.W., Weaver V.M., Calderwood D.A., Keely P.J. (2009). Filamin A-beta1 integrin complex tunes epithelial cell response to matrix tension. Mol. Biol. Cell.

[150] Mammoto A., Huang S., Ingber D.E. (2007). Filamin links cell shape and cytoskeletal structure to Rho regulation by controlling accumulation of p190RhoGAP in lipid rafts. J. Cell Sci..

[151] Boyd N., Guo H., Martin L.J., Sun L., Stone J., Fishell E., Jong R.A., Hislop G., Chiarelli A., Minkin S. (2007). Mammographic density and the risk and detection of breast cancer. N. Engl. J. Med..

[152] Pollan M., Ascunce N., Ederra M., Murillo A., Erdozáin N., Alés-Martínez J.E., Pastor-Barriuso R. (2013). Mammographic density and risk of breast cancer according to tumor characteristics and mode of detection: A Spanish population-based case-control study. Breast Cancer Res..

[153] Nazari S.S., Mukherjee P. (2018). An overview of mammographic density and its association with breast cancer. Breast Cancer.

[154] Guo Y.P., Martin L.J., Hanna W., Banerjee D., Miller N., Fishell E., Khokha R., Boyd N.F. (2001). Growth factors and stromal matrix proteins associated with mammographic densities. Cancer Epidemiol. Biomark. Prev..

[155] Alowami S., Troup S., Al-Haddad S., Kirkpatrick I., Watson P.H. (2003). Mammographic density is related to stroma and stromal proteoglycan expression. Breast Cancer Res..

[156] Provenzano P.P., Inman D.R., Eliceiri K.W., Keely P.J. (2009). Matrix density-induced mechanoregulation of breast cell phenotype, signaling and gene expression through a FAK–ERK linkage. Oncogene.

[157] Provenzano P.P., Inman D.R., Eliceiri K.W., Knittel J.G., Yan L., Rueden C., White J., Keely P.J. (2008). Collagen density promotes mammary tumor initiation and progression. BMC Med..

[158] Neuhouser M.L., Aragaki A.K., Prentice R.L., Manson J.E., Chlebowski R., Carty C.L., Ochs-Balcom H.M., Thomson C.A., Caan B.J., Tinker L.F. (2015). Overweight, obesity, and postmenopausal invasive breast cancer risk: A secondary analysis of the women’s health initiative randomized clinical trials. JAMA Oncol..

[159] Strong A.L., A Strong T., Rhodes L., A Semon J., Zhang X., Shi Z., Zhang S., Gimble J.M., Burow M.E., Bunnell B.A. (2013). Obesity associated alterations in the biology of adipose stem cells mediate enhanced tumorigenesis by estrogen dependent pathways. Breast Cancer Res..

[160] Soguel L., Durocher F., Tchernof A., Diorio C. (2017). Adiposity, breast density, and breast cancer risk: Epidemiological and biological considerations. Eur. J. Cancer Prev..

[161] Shoham N., Gefen A. (2012). Mechanotransduction in adipocytes. J. Biomech..

[162] Boyd N., Li Q., Melnichouk O., Huszti E., Martin L.J., Gunasekara A., Mawdsley G., Yaffe M.J., Minkin S. (2014). Evidence that breast tissue stiffness is associated with risk of breast cancer. PLoS ONE.

[163] Chen J.H., Chan S., Zhang Y., Li S., Chang R.-F., Su M.-Y. (2019). Evaluation of breast stiffness measured by ultrasound and breast density measured by MRI using a prone-supine deformation model. Biomark. Res..

[164] Huang L., Ma M., Du Z., Liu Z., Gong X. (2019). Quantitative evaluation of tissue stiffness around lesion by sound touch elastography in the diagnosis of benign and malignant breast lesions. PLoS ONE.

[165] Choi W.J., Kim H.H., Cha J.H., Shin H.J., Kim H., Chae E.Y., Hong M.J. (2014). Predicting prognostic factors of breast cancer using shear wave elastography. Ultrasound Med. Biol..

[166] Evans A., Whelehan P., Thomson K., McLean D., Brauer K., Purdie C., Baker L., Jordan L., Rauchhaus P., Thompson A. (2012). Invasive breast cancer: Relationship between shear-wave elastographic findings and histologic prognostic factors. Radiology.

[167] Acerbi I., Cassereau L., Dean I., Shi Q., Au A., Park C., Chen Y.Y., Liphardt J., Hwang E.S., Weaver V.M. (2015). Human breast cancer invasion and aggression correlates with ECM stiffening and immune cell infiltration. Integr. Biol. (Camb.).

[168] López J.I., Kang I., You W.-K., McDonald N.M., Weaver V.M. (2011). In situforce mapping of mammary gland transformation. Integr. Biol..

[169] Tanter M., Bercoff J., Athanasiou A., Deffieux T., Gennisson J.-L., Montaldo G., Muller M., Tardivon A., Fink M. (2008). Quantitative assessment of breast lesion viscoelasticity: Initial clinical results using supersonic shear imaging. Ultrasound Med. Biol..

[170] Zhou J., Zhan W., Chang C., Zhang X., Jia Y., Dong Y., Zhou C., Sun J., Grant E.G. (2014). Breast lesions: Evaluation with shear wave elastography, with special emphasis on the “Stiff Rim” sign. Radiology.

[171] Ren W.-W., Li X.-L., He Y.-P., Li D.-D., Wang D., Zhao C.-K., Bo X.-W., Liu B.-J., Yue W.-W., Xu H.-X. (2017). Two-dimensional shear wave elastography of breast lesions: Comparison of two different systems. Clin. Hemorheol. Microcirc..

[172] Evans A., Whelehan P., Thomson K., McLean D., Brauer K., Purdie C., Jordan L.B., Baker L., Thompson A. (2010). Quantitative shear wave ultrasound elastography: Initial experience in solid breast masses. Breast Cancer Res..

[173] Tozaki M., Fukuma E. (2011). Pattern classification of ShearWaveTM Elastography images for differential diagnosis between benign and malignant solid breast masses. Acta Radiol..

[174] Youk J.H., Gweon H.M., Son E.J. (2017). Shear-wave elastography in breast ultrasonography: The state of the art. Ultrasonography.

[175] Moon J.H., Hwang J.Y., Park J.S., Koh S.H., Park S.Y. (2018). Impact of region of interest (ROI) size on the diagnostic performance of shear wave elastography in differentiating solid breast lesions. Acta Radiol..

[176] Chaudhuri O., Koshy S., Da Cunha C.B., Shin J.-W., Verbeke C.S., Allison K.H., Mooney D.J. (2014). Extracellular matrix stiffness and composition jointly regulate the induction of malignant phenotypes in mammary epithelium. Nat. Mater..

[177] Ondeck M.G., Kumar A., Placone J.K., Plunkett C.M., Matte B.F., Wong K.C., Fattet L., Yang J., Engler A.J. (2019). Dynamically stiffened matrix promotes malignant transformation of mammary epithelial cells via collective mechanical signaling. Proc. Natl. Acad. Sci. USA.

[178] Stowers R.S., Allen S.C., Sanchez K., Davis C.L., Ebelt N., Berg C.V.D., Suggs L.J. (2016). Extracellular matrix stiffening induces a malignant phenotypic transition in breast epithelial cells. Cell. Mol. Bioeng..

[179] Young J.S., Llumsden C.E., Stalker A.L. (1950). The significance of the “tissue pressure” of normal testicular and of neoplastic (Brown-Pearce carcinoma) tissue in the rabbit. J. Pathol. Bacteriol..

[180] Boucher Y., Jain R.K. (1992). Microvascular pressure is the principal driving force for interstitial hypertension in solid tumors: Implications for vascular collapse. Cancer Res..

[181] Less J.R., Posner M.C., Boucher Y., Borochovitz D., Wolmark N., Jain R.K. (1992). Interstitial hypertension in human breast and colorectal tumors. Cancer Res..

[182] Nathanson S.D., Nelson L. (1994). Interstitial fluid pressure in breast cancer, benign breast conditions, and breast parenchyma. Ann. Surg. Oncol..

[183] Boucher Y., Baxter L.T., Jain R.K. (1990). Interstitial pressure gradients in tissue-isolated and subcutaneous tumors: Implications for therapy. Cancer Res..

[184] Hobbs S.K., Monsky W.L., Yuan F., Roberts W.G., Griffith L.G., Torchilin V.P., Jain R.K. (1998). Regulation of transport pathways in tumor vessels: Role of tumor type and microenvironment. Proc. Natl. Acad. Sci. USA.

[185] Griffon-Etienne G., Boucher Y., Brekken C., Suit H.D., Jain R.K. (1999). Taxane-induced apoptosis decompresses blood vessels and lowers interstitial fluid pressure in solid tumors: Clinical implications. Cancer Res..

[186] Milosevic M., Fyles A., Hedley D., Pintilie M., Levin W., Manchul L., Hill R. (2001). Interstitial fluid pressure predicts survival in patients with cervix cancer independent of clinical prognostic factors and tumor oxygen measurements. Cancer Res..

[187] Baxter L.T., Jain R.K. (1989). Transport of fluid and macromolecules in tumors. I. Role of interstitial pressure and convection. Microvasc. Res..

[188] Butler T.P., Grantham F.H., Gullino P.M. (1975). Bulk transfer of fluid in the interstitial compartment of mammary tumors. Cancer Res..

[189] Chary S.R., Jain R.K. (1989). Direct measurement of interstitial convection and diffusion of albumin in normal and neoplastic tissues by fluorescence photobleaching. Proc. Natl. Acad. Sci. USA.

[190] Dafni H., Israely T., Bhujwalla Z.M., E Benjamin L., Neeman M. (2002). Overexpression of vascular endothelial growth factor 165 drives peritumor interstitial convection and induces lymphatic drain: Magnetic resonance imaging, confocal microscopy, and histological tracking of triple-labeled albumin. Cancer Res..

[191] Tang K., Li S., Li P., Xia Q., Yang R., Li T., Li L., Jiang Y., Qin X., Yang H. (2020). Shear stress stimulates integrin beta1 trafficking and increases directional migration of cancer cells via promoting deacetylation of microtubules. Biochim. Biophys. Acta Mol. Cell Res..

[192] Piotrowski-Daspit A.S., Tien J., Nelson C.M. (2016). Interstitial fluid pressure regulates collective invasion in engineered human breast tumors via Snail, vimentin, and E-cadherin. Integr. Biol..

[193] Novak C.M., Horst E.N., Taylor C.C., Liu C.Z., Mehta G. (2019). Fluid shear stress stimulates breast cancer cells to display invasive and chemoresistant phenotypes while upregulating PLAU in a 3D bioreactor. Biotechnol. Bioeng..

[194] Shieh A., Rozansky H.A., Hinz B., Swartz M.A. (2011). Tumor cell invasion is promoted by interstitial flow-induced matrix priming by stromal fibroblasts. Cancer Res..

[195] Polacheck W., Charest J.L., Kamm R.D. (2011). Interstitial flow influences direction of tumor cell migration through competing mechanisms. Proc. Natl. Acad. Sci. USA.

[196] Nia H.T., Datta M., Seano G., Huang P., Munn L.L., Jain R.K. (2018). Quantifying solid stress and elastic energy from excised or in situ tumors. Nat. Protoc..

[197] Helmlinger G., Netti P., Lichtenbeld H.C., Melder R.J., Jain R.K. (1997). Solid stress inhibits the growth of multicellular tumor spheroids. Nat. Biotechnol..

[198] Cheng G., Tse J., Jain R.K., Munn L. (2009). Micro-environmental mechanical stress controls tumor spheroid size and morphology by suppressing proliferation and inducing apoptosis in cancer cells. PLoS ONE.

[199] Stylianopoulos T., Martin J.D., Chauhan V., Jain S.R., Diop-Frimpong B., Bardeesy N., Smith B.L., Ferrone C.R., Hornicek F.J., Boucher Y. (2012). Causes, consequences, and remedies for growth-induced solid stress in murine and human tumors. Proc. Natl. Acad. Sci. USA.

[200] Padera T.P., Stoll B.R., Tooredman J.B., Capen D., di Tomaso E., Jain R.K. (2004). Pathology: Cancer cells compress intratumour vessels. Nature.

[201] Chauhan V., Martin J.D., Liu H., Lacorre D.A., Jain S.R., Kozin S.V., Stylianopoulos T., Mousa A.S., Han X., Adstamongkonkul P. (2013). Angiotensin inhibition enhances drug delivery and potentiates chemotherapy by decompressing tumour blood vessels. Nat. Commun..

[202] Stylianopoulos T., Martin J.D., Snuderl M., Mpekris F., Jain S.R., Jain R.K. (2013). Coevolution of solid stress and interstitial fluid pressure in tumors during progression: Implications for vascular collapse. Cancer Res..

[203] Fovargue D., Fiorito M., Capilnasiu A., Nordsletten D., Lee J., Sinkus R. (2020). Towards noninvasive estimation of tumour pressure by utilising MR elastography and nonlinear biomechanical models: A simulation and phantom study. Sci. Rep..

[204] Fernandez-Sanchez M.E., Barbier S., Whitehead J., Bealle G., Michel A., Latorre-Ossa H., Rey C., Fouassier L., Claperon A., Brulle L. (2015). Mechanical induction of the tumorigenic beta-catenin pathway by tumour growth pressure. Nature.

[205] Seano G., Nia H.T., Emblem K.E., Datta M., Ren J., Krishnan S., Kloepper J., Pinho M.C., Ho W.W., Ghosh M. (2019). Solid stress in brain tumours causes neuronal loss and neurological dysfunction and can be reversed by lithium. Nat. Biomed. Eng..

[206] Tse J.M., Cheng G., Tyrrell J.A., Wilcox-Adelman S.A., Boucher Y., Jain R.K., Munn L.L. (2011). Mechanical compression drives cancer cells toward invasive phenotype. Proc. Natl. Acad. Sci. USA.

[207] Shi Q., Ghosh R., Engelke H., Rycroft C.H., Cassereau L., Sethian J.A., Weaver V.M., Liphardt J.T. (2013). Rapid disorganization of mechanically interacting systems of mammary acini. Proc. Natl. Acad. Sci. USA.

[208] Ricca B.L., Venugopalan G., Furuta S., Tanner K., Orellana W.A., Reber C.D., Brownfield D.G., Bissell M.J., Fletcher D. (2018). Transient external force induces phenotypic reversion of malignant epithelial structures via nitric oxide signaling. eLife.

[209] Indra I., Gasparski A.N., Beningo K.A. (2018). An in vitro correlation of metastatic capacity and dual mechanostimulation. PLoS ONE.

[210] Xu J., Mathur J., Vessieres E., Hammack S., Nonomura K., Favre J., Grimaud L., Petrus M., Francisco A., Li J. (2018). GPR68 senses flow and is essential for vascular physiology. Cell.

[211] Wiley S.Z., Sriram K., Salmerón C., Insel P.A. (2019). GPR68: An emerging drug target in cancer. Int. J. Mol. Sci..

[212] Scholz N. (2018). Cancer cell mechanics: Adhesion G protein-coupled receptors in action?. Front. Oncol..

[213] Tang X., Jin R., Qu G., Wang X., Li Z., Yuan Z., Zhao C., Siwko S., Shi T., Wang P. (2013). GPR116, an adhesion G-protein-coupled receptor, promotes breast cancer metastasis via the Galphaq-p63RhoGEF-Rho GTPase pathway. Cancer Res..

[214] Zhu C., Chen W., Lou J., Rittase W., Li K. (2019). Mechanosensing through immunoreceptors. Nat. Immunol..

[215] Hu K.H., Butte M.J. (2016). T cell activation requires force generation. J. Cell Biol..

[216] Basu R., Whitlock B.M., Husson J., Le Floc’H A., Jin W., Oyler-Yaniv A., Dotiwala F., Giannone G., Hivroz C., Biais N. (2016). Cytotoxic T cells use mechanical force to potentiate target cell killing. Cell.

[217] Saitakis M., Dogniaux S., Goudot C., Bufi N., Asnacios S., Maurin M., Randriamampita C., Asnacios A., Hivroz C. (2017). Different TCR-induced T lymphocyte responses are potentiated by stiffness with variable sensitivity. eLife.

[218] Wan Z., Zhang S., Fan Y., Liu K., Du F., Davey A.M., Zhang H., Han W., Xiong C., Liu W. (2013). B cell activation is regulated by the stiffness properties of the substrate presenting the antigens. J. Immunol..

[219] Balzer E.M., Whipple R.A., Cho E., Matrone M.A., Martin S. (2009). Antimitotic chemotherapeutics promote adhesive responses in detached and circulating tumor cells. Breast Cancer Res. Treat..

[220] Chakrabarti K.R., Hessler L., Bhandary L., Martin S. (2015). Molecular pathways: New signaling considerations when targeting cytoskeletal balance to reduce tumor growth. Clin. Cancer Res. Off. J. Am. Assoc. Cancer Res..

[221] Khairallah R.J., Shi G., Sbrana F., Prosser B.L., Borroto C., Mazaitis M.J., Hoffman E.P., Mahurkar A., Sachs F., Sun Y. (2012). Microtubules underlie dysfunction in duchenne muscular dystrophy. Sci. Signal..

